# Profiles of epistemological beliefs, knowledge about explanation norms, and explanation skills: changes after an intervention

**DOI:** 10.3389/fpsyg.2023.1178129

**Published:** 2023-10-20

**Authors:** Eric Klopp, Theresa Krause-Wichmann, Robin Stark

**Affiliations:** Department of Education, Saarland University, Saarbrücken, Germany

**Keywords:** epistemological beliefs, scientific explanations, intervention, epistemological belief change, latent profile transition analysis

## Abstract

In this study, we exploratively investigate the relation between students’ epistemological beliefs and their declarative knowledge about scientific explanations and their practical skills to explain psychological phenomena drawing on scientific theories before and after a training intervention using a person-centered approach. We theoretically derive profiles of epistemological beliefs that should be beneficial for constructing scientific explanations. We those having higher explanation skills show a profile of epistemological beliefs that is beneficial for explanations skills. Using a latent profile transition analysis and a sample with *N* = 108 students, we explore which profiles of epistemological beliefs, declarative knowledge about explanations, and explanation skills empirically emerge before and after an intervention that aimed and fostering students’ skills to construct scientific explanations. Before the intervention, two profiles emerged that differed in epistemological beliefs and explanation skills, but both did not in declarative knowledge about explanation. The intervention, in general, yielded a gain in declarative knowledge about explanations and explanation skills. After the intervention, again, two profiles emerged. However, these profiles did not differ in their epistemological beliefs but only in declarative knowledge about explanations and explanation skills. Thus, the intervention seems to level out the effects of epistemological beliefs. Additionally, the pattern of change in epistemological beliefs is consistent with theoretical expectations about which epistemological beliefs are beneficial for explanations. We discuss the results and their implications, as well as their limitations. Finally, we provide an outlook of using the person-oriented approach and this study’s type of intervention in the research on changing epistemological beliefs.

## 1. Introduction

A major issue in psychology is the construction of scientific explanations of observable behavior using psychological theories (e.g., Gerrig and Zimbardo, 2010). Therefore, being able to create scientific explanations is a core component of scientific competencies in psychology (Dietrich et al., 2015) and a part of scientific thinking in this domain. This applies to psychology and disciplines in which psychology plays a major role, like education. For instance, in Germany, the Standing Conference of the Ministers of Education and Cultural Affairs of the States (2019) requires teachers and teacher students to be able to explain teaching and learning processes drawing on knowledge from the educational sciences, in particular from educational psychology.

For instance, observing a pupil’s case of declining grades in mathematics in combination with some physiological symptoms may be explained using the theory of test anxiety (cf., Zeidner, 1998). Such an explanation provides a causal account of why the pupil’s test anxiety emerged and possible indications for interventions.

However, using theories from educational psychology’s body of knowledge to generate explanations of teaching and learning processes requires a reflective thinking process. It is widely known that epistemological beliefs are related to such reflective thinking processes. Epistemological beliefs are a person’s subjective notions about knowledge and the process of knowledge acquisition. They are important predictors for the quality of reflective thinking processes (e.g., Fischer et al., 2014) and thus may play an important role in the ability to construct a scientific explanation.

Typically, the relation between epistemological beliefs and other variables is investigated using a variable-centered approach, i.e., the relation between variables is of interest disregarding the individual. However, a person-oriented approach has recently been introduced to the research on epistemological beliefs (e.g., Kampa et al., 2016; Schiefer et al., 2022). The person-oriented approach focuses on the individual as the unit of analysis on the level of phenomena (cf., Bergman et al., 2003). It comprises its own methodological considerations and methods. Hereby methods are used that typically try to find groups of people with the same characteristics in a set of variables that can be expressed in the form of profiles.

In the present explorative study, we investigate the role of epistemological beliefs in constructing scientific explanations. In particular, we investigate how epistemological beliefs and the ability to construct scientific explanations relate before and after an intervention to foster students’ explanation skills from a person-centered perspective. In particular, we investigate if there are profiles of epistemological beliefs and explanation skills that can be identified before and how the profiles change after the intervention. We use a latent profile transition analysis, allowing us to scrutinize patterns of changes that are, e.g., introduced by events like interventions (cf., Hickendorff et al., 2018).

In the following section 1.1, we firstly introduce the concept of scientific explanations and describe the structure and the norms of explanations. We also introduce the concept of explanations skills as the competency to construct scientific explanations and lay out how students’ explanation skills can be fostered. Section 1.2 introduces epistemological beliefs by characterizing the two main approaches to the field. Combining these two approaches into a common framework raises the idea of epistemological belief profiles. We also describe how these profiles develop throughout the socialization with scientific concepts like explanations. To end this section, we describe how certain epistemological belief dimensions relate to explanations skills. In section 1.3, we outline the person-centered approach and its methodology, and finally, in section 1.4, we present the research questions of this study. Afterward, we present and discuss the results and describe possible implications for research and education.

### 1.1. The concept of scientific explanations and methods to foster explanation skills

**Figure 1:**
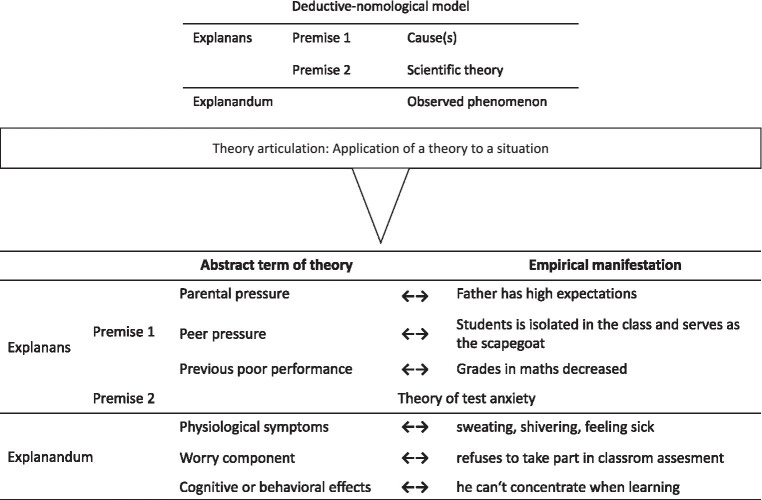
The structure of an explanation according to the DN-model (upper part) and the concept of theory articulation as the application of a theory to a situation, in particular, the mapping between the abstract terms of the theory with the empirically observable elements of the situation.

According to Ohlsson (2010), scientific explanations provide an answer to questions of why a certain (psychological) phenomenon happened, e.g., why a student developed test anxiety. In this way, explanations provide a means to construct a causal model of the observed phenomenon (cf., Kim, 1994). Explanations follow a given structure, and perhaps the most well-known structural model for explanations is the deductive-nomological model^1^ (hereafter: DN model; Hempel and Oppenheim, 1948). The DN model consists of two components (Figure 1): the explanandum and the explanans. The explanandum contains the phenomenon to be explained, whereas the explanans consists of a set of sentences that are stated to account for the phenomenon. The first sentence in the explanans is a theory mentioning the causes of the phenomenon at hand. This first sentence is also called the explanation’s first premise. The second sentence contains a statement that mentions that the causes stated in the first sentence’s theory are present in the observed phenomenon. This second sentence is also called the explanation’s second premise. The explanandum is then logically deduced from the two premises.

Ohlsson’s (1992) concept of theory articulation states that an explanation is the application of a scientific theory to a situation by mapping a theory onto a situation. The mapping relates the theoretical causes and consequences stated in the abstract terms of a theory to the observed terms of the situation. The main point in an explanation’s application is the elaboration of how the abstract terms of the theory manifest in the situation. Such an elaboration of the relation between a theory and a given phenomenon is the basis for a scientific understanding of the phenomenon (cf., McCain, 2015). The following case example illustrates the structure and the mapping between the abstract terms of the theory and their empirical manifestation:

“Peter’s math grades got worse during the previous year. The math teacher observes that Peter is nervous before each math assessment: He is sweating and shivering and says he is feeling sick. Moreover, he refuses to take part in classroom assessments because he cannot concentrate on learning and worries about his performance. The math teacher talks with Peter’s father about the issues. The father is a lawyer and has high expectations because he wants Peter to become a lawyer, too. Additionally, the math teacher observes that Peter is mostly alone, isolated from the other students, and often serves as a scapegoat in the class.”

**Figure 2:**
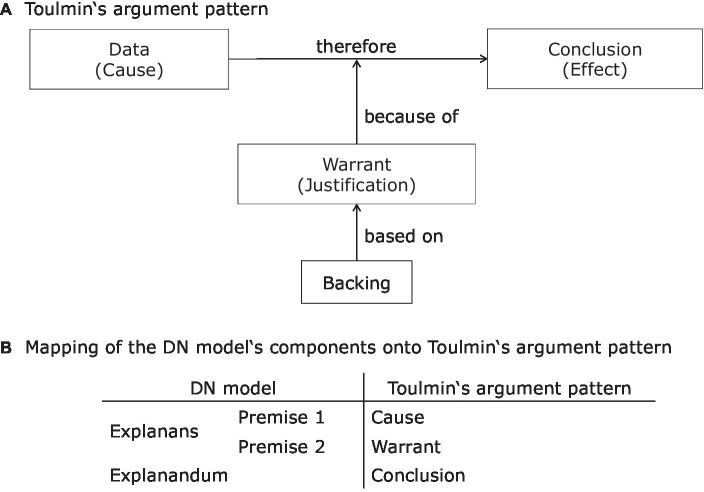
Toulmin’s (2003) argument pattern **(A)** and it’s relation to the DN model of scientific explanations **(B)**.

In this situation, the theory of test anxiety (cf., Zeidner, 1998) can account for Peter’s behavior. The theory states that parental pressure, peer pressure, and previous poor performance can result in physiological symptoms, like worries, and cognitive or behavioral consequences. Figure 1 illustrates the DN model as an explanation’s basic structure in the upper part and, secondly, the conception of theory articulation in the lower part. The lower part illustrates the mapping between the abstract terms of the theory and the empirical manifestations in the observed situation. Scientific explanations are structurally related to arguments. In terms of Toulmin’s (2003) argument pattern, a basic argument consists of three parts: The first part is called data (sometimes called cause) and consists of empirical observations. The second part is called warrant (sometimes called justification) and consists of a scientific assertion. From the combination of data and warrant, a conclusion (sometimes called effect) is derived. Put differently, the combination of warrant and the data provide the reasons for the conclusion. Finally, there is a backing which acts as a justification of the warrant. The structure of this argument pattern is shown in the upper panel of Figure 2. Toulmin’s argument pattern can be mapped onto the structure of the DN model: the first and second premise corresponds to the data and the warrant, whereas the explanandum corresponds to the conclusion, this mapping is depicted in the lower panel of Figure 2. Thus, an explanation is a special case of an argument.

Constructing an explanation is a form of deductive reasoning in which theories and the observed phenomena must be coordinated (cf., Tytler and Peterson, 2003). This deductive inference must be logically correct and thus logical correctness is a norm for a valid explanation (Rosenberg, 2012). Logical correctness is the first norm to which a skilled explanation must adhere. As the explanandum constitutes a deduction from the two parts of the explanans, an explanation must not contain circular reasoning (Woodward, 2014), which constitutes a second norm. In addition to this norm, valid explanations must comply with several other norms (Westermann, 2000; Rosenberg, 2012). The third norm states that scientific explanations must draw on empirically proved theories and therefore prevent using non-scientific every day or subjective theories (cf., Stark, 2005). As a fourth norm, the theory must also be explicitly mentioned. A fifth norm requires a detailed elaboration of the relationship between the theory and the situation, hereafter theory-evidence-coordination. This norm draws on the distinction between theories and evidence (Kuhn, 1997) and refers to the ability to relate the abstract terms of a theory to their manifestation in the observed situation and to decide which available theory fits the observed phenomenon best. This norm represents the mapping of the theory’s abstract terms onto the situation’s observed terms in Ohlsson’s (1992) concept of theory articulation and furthermore relates to appropriate recognition which piece of evidence relates to which piece of the theory, or which piece of evidence in indicative for a certain theory. A logically correct explanation requires a proper mapping between the antecedent condition and the phenomenon in the sense of its respective empirical manifestation. Circular reasoning, which would violate the first norm, would be evident if the same empirical observations were mentioned both in the antecedent condition and in the phenomenon. The sixth and final norm refers to considering alternative theories that can either explain the situation, can only explain part of the situation, or cannot explain the situation at all. This norm draws on argumentation theory (Walton, 1989), and we call it multiperspectivity (cf., Klopp and Stark, 2018). Since explanations are typically used in scientific argumentative discourse, it is important to protect or support one’s argument, as represented by the explanation, by either mentioning other potentially relevant theories or ruling out theories that do not fit the observed phenomenon. The sixth norm again highlights the relation between an argument and an explanation. This norm represents the backing part of Toulmin’s (2003) argument pattern as this norm provides a justification for the chosen theory.

In the context of scientific competencies, Dietrich et al. (2015) conceptualize the skill to construct an explanation as a cognitive process that enables a person to construct a causal model that shows how an observable psychological phenomenon, e.g., test anxiety, can be understood by drawing on psychological theories (cf., Kim, 1994; Klopp and Stark, 2018). Additionally, following Anderson et al.’s (2001) distinction between cognitive processes and knowledge, explanation skills require declarative knowledge about the structure of explanations and the norms of scientifically valid explanations.

There are several methods to foster explanations skills. Learning from worked examples is especially suited for initial knowledge acquisition in well-structured domains that depend on certain structures and rules. It involves studying and analyzing a step-by-step demonstration of how to solve a task. A worked example provides expert problem-solving strategies and learners can observe the correct application of structures and rules. The learner’s task is to elaborate on the worked example. As a worked example contains the solution, the learners are able to focus their attention towards understanding the solution and the problem-solving steps allowing them to construct a mental representation of the problem-solving process and develop a schema for solving similar problems in the future (cf., Renkl, 2014). To foster explanation skills, students receive examples of valid explanations demonstrating the structure and the applications of the norms of scientific explanations. Klopp and Stark (2018) showed that learning with worked examples can foster psychology students’ explanation skills and declarative knowledge about explanations’ structure and norms.

Another potentially effective instructional method is learning from advocatory errors (Oser, 2007; Oser et al., 2012). Errors are generally considered as deviations from a given norm (Mehl, 1993). As explanation skills consist mainly in applying the explanation norms, learning from advocatory errors may be an effective way to foster explanation skills. In learning from advocatory errors, learners acquire knowledge when observing the errors of relevant others in the social environment. Learning occurs when the learner contrasts the error with the correct solution (Wagner et al., 2014a). Learning from advocatory errors yields negative knowledge. Negative knowledge is knowledge about what is wrong and what must be avoided during task performance (Gartmeier et al., 2008). Finally, avoidance strategies, i.e., strategies to avoid the error in future task performances, are an integral part of negative knowledge. Oser (2007) extends the social environment to include fictive actions, e.g., stories, novels, movies, etc. To be effective, the learner has to be aware of the error, understand the error, have the motivation to correct the error and must be presented with the correct solution. Additionally, the learners must identify with the person committing the error, and the context in which the error occurs should also be relevant for the learners (Oser, 2007). Wagner et al. (2014a,b), Klein et al., (2017), and Klopp and Stark, (2020) showed that learning from advocatory errors effectively fostered student teachers’ skills to explain authentic school situations using scientific theories.

### 1.2. Epistemological beliefs: their structure and relation to explanation skills

Epistemological beliefs are important for argumentation skills (cf., Fischer et al., 2014). In the extensive work of Kuhn (e.g., Kuhn, 1991, 1997, 2001), she provided a large body of evidence that evaluativist epistemological beliefs are adequate for constructing proper scientific arguments. Therefore, in terms of the integrated approach, certain profiles of epistemological belief dimensions are more favorable for proper argumentation than others. Since explanations are special cases of scientific arguments as they share the same structure as shown in the preceding section, and as explanations are within the realm of scientific thinking, epistemological beliefs are also an important factor determining an individual’s ability to construct a scientific explanation. Additionally, epistemological beliefs may determine which body of knowledge, e.g., theories or concepts, are considered scientific. Guilfoyle et al. (2020) found that epistemological beliefs may affect the acceptance of educational research in initial teacher education. As scientific explanations draw on the use of scientific theories, given Guilfoyle et al.’s (2020) results, epistemological beliefs are likely important for the choice of the theories used in explanations, too. For instance, individuals with a profile of epistemological beliefs indicating a disregard for educational research may resort to everyday theories instead of scientific theories, which violates the norm that scientific explanations must draw on scientific theories. Thus, an inadequate epistemological belief may yield an erroneous explanation. In the following, we briefly outline the two common approaches to epistemological beliefs and how they can be integrated into a common framework. Afterward, we describe how external factors, e.g., interventions, yield a change of the epistemological belief profiles.

There are two broad approaches to this concept. In the beliefs approach of Hofer and Pintrich (1997), epistemological beliefs are considered dimensions of interindividual differences. These authors propose the four dimensions Certainty of knowledge, Simplicity of knowledge, Source of knowledge, and Justification of knowing. In contrast, in the developmental approach, epistemological beliefs are described by the three sequential levels of epistemological development, i.e., absolutism, multiplicism, and evaluativism. Recently, the beliefs and the developmental approach have been integrated into a common framework (Weinstock, 2006; Greene et al., 2008, 2010). In this integrated approach, interindividual differences in beliefs and levels of development are not distinct constructs but two sides of the same coin. For instance, Weinstock (2006) characterizes each of the three developmental levels as a certain profile of the four epistemological beliefs dimensions proposed by Hofer and Pintrich (1997). A profile means a certain configuration of several epistemological belief dimensions; in this case, the four dimensions from Hofer and Pintrich (1997). However, the integrated approach is not restricted to the dimensions of Hofer and Pintrich (1997). For instance, Barzilai and Weinstock (2015) summarized the most common epistemological belief dimensions from the current literature and worked out profiles that characterize the three developmental levels. Thus, in the most current approach to the development of epistemological beliefs, they can be modeled as profiles of dimensions of interindividual differences.

Epistemological beliefs are typically acquired and develop during the enculturation in a domain (Palmer and Marra, 2008; Klopp and Stark, 2018). Palmer and Marra’s (2008) ecological model of personal epistemologies describes the effects of the direct and indirect environment on the development of epistemological beliefs. The direct environment consists of lectures, seminars, and other instructional instances, and the indirect environment refers to various domains and scientific institutions to which students are exposed. Exposure to these different kinds of environments yields changes in epistemological beliefs. The Process Model of Personal Epistemology Development (Bendixen, 2002; see also Bendixen and Rule, 2004) describes the necessary cognitive mechanisms to induce changes in epistemological beliefs. The model postulates three mechanisms: epistemological doubt, epistemological volition, and resolution strategies. Epistemological doubt refers to questioning one’s current epistemological beliefs due to a dissonance between current beliefs and a new experience. Epistemological volition refers to a concentrated effort to change the current epistemological beliefs to the affordances and constraints of the new experience. Resolution strategies describe how epistemological beliefs are altered. A prominent resolution strategy is reflection. Reflection involves reviewing past experiences and one’s current epistemological beliefs and analyzing implications. If all these components interact, epistemological change is induced. The notion of epistemological change in the integrated approach to epistemological beliefs can be thought of as the asynchronous change in individual profiles of epistemological belief dimensions (Schommer-Aikins, 2002). Thus, epistemological change, e.g., caused by exposure to elements of the direct environment like seminars or lectures on specific topics, can be understood as a change in the related profiles.

Epistemological beliefs are deeply related to scientific thinking (cf., Fischer et al., 2014). An important dimension of scientific thinking encompasses the cognitive processes necessary to construct a scientific explanation. As described in the previous section, an explanation follows a certain structure and certain norms and uses a scientific theory to justify the occurrence of an observable phenomenon. This corresponds to the construction of an argument, which also has to follow a corresponding structure and norms. Thus, several epistemological belief dimensions are related to either the norms of explanations or scientific theories that may affect explanation skills or the acquisition of explanation skills.

In the following, we present a synthesis of the most common epistemological belief dimensions that are theoretically related to the concept of scientific explanations. For instance, the dimension of Personal justification (Greene et al., 2010) describes interindividual differences in the belief that scientific knowledge consists merely of the personal opinion of scientists. It also entails the belief that scientific authorities disseminate their own opinion as a scientific fact. A firm belief in Personal justification may hinder the use of scientific theories in explanations because scientific theories are potentially disregarded as a scientist’s opinion, and everyday-theories or folk theories are used to construct an explanation, because they are erroneously believed to have the same quality. Thus, this dimension is directly relevant to the norm of using scientific theories.

The dimension Justification by authority (Greene et al., 2010) describes interindividual differences in the tendency to trust scientific authorities like scientists or other sources like textbooks. It also entails the tendency to trust knowledge claims that originate from a specific scientific source, e.g., a particular scientist or scientific domain. A firm belief in Justification by authority may also hinder the adequate use of scientific theories. Additionally, individuals with a firm belief may also favor a certain domain when they ascribe a certain level of authority to this domain in general, regardless of whether the domain’s theories refer to the current situation. For instance, individuals may prefer theories from neuroscience in general, regardless of whether they relate to the current issue. An example at hand would be that an inappropriate neuroscientific theory is used to construct an explanation for the case of test anxiety because the explainer has high trust in an authoritative figure promoting this theory, regardless of whether this figure is a domain expert in test anxiety or not.

Justification by multiple sources (Braten et al., 2014) describes interindividual differences in the belief that there must be multiple sources to corroborate a knowledge claim like a scientific theory and that there is the necessity to check multiple sources in the verification process of a knowledge claim. Concerning the multiperspectivity in explanations, individuals with a firm belief may consider alternative theories – either in the sense of their exclusion, in the sense of real alternatives, or in the sense of theories that partially fit a situation – because those individuals are more likely to consult several sources.

The dimension Justification by the scientific community (cf., Moschner and Gruber, 2017) describes interindividual differences in the belief that the scientific community must recognize a scientific theory to count as valid knowledge. Therefore, a firm belief in the Justification by the scientific community may be adequate for constructing an explanation because the explainer is more likely to select a scientific theory in an explanation in contrast to everyday-theories or folk theories. Additionally, an individual with a firm belief may prefer proven theories in contrast to theories under scrutiny.

The commonality of the previous four dimensions of epistemological beliefs is that they refer directly to the nature of scientific knowledge, particularly the conditions that must hold for valid scientific knowledge. But other kinds of epistemological beliefs may relate to scientific explanations in general and explanation skills in particular. The dimension Certainty of knowledge (Moschner and Gruber, 2017) describes interindividual differences in the belief that scientists can come to the final truth and that there may be knowledge that holds forever. The dimension Reflective nature of knowledge (Moschner and Gruber, 2017) represents interindividual differences in the belief that knowledge may be revised depending on new insights and that new experiences may alter the current knowledge base. Certainty of knowledge and Reflective nature of knowledge may affect if and to which degree individuals are willing to reflect on their previous conceptions of explanations when they get introduced to the concept of scientific explanations, either in the context of socialization in science or in specific training interventions. In particular, a weak belief in the Certainty of knowledge and a firm belief in the Reflective nature of knowledge may affect how much an individual is willing to reflect on their previous conceptions of explanations.

To sum up, there may be a profile of epistemological beliefs that is adequate for explanation skills. Adequate is a term that was introduced in text context of epistemological beliefs by Klopp and Stark (2022a) and means that a certain profile of epistemological beliefs is satisfactory in their quality to cope with the knowledge structure in a certain domain. In the context of the current study, the term adequate can thus be applied in the sense that there is a certain profile of epistemological beliefs that is satisfactory in their quality to cope with the affordances of scientific explanations.^2^ Put differently, adequate means that a certain epistemological belief profile creates behaviors and habits (cf., Elby and Hammer, 2001) leading to the successful achievement of explanations skills that, in turn, result in the skilled construction of explanations according to norms for valid scientific explanations. Following the argumentation above, it is reasonable to expect that the following epistemological belief profile is adequate for explanation skills:

- Personal justification/Justification by authority: weak beliefs
- Justification by multiple sources/Justification by the scientific community: strong beliefs
- Certainty of knowledge: weak belief
- Reflective nature of knowledge: strong beliefs

Such a profile of adequate epistemological beliefs may likely be acquired in the scientific socialization process when there is a transition from a naïve concept of explanation to a scientific concept and a transition from everyday epistemological beliefs to more scientific-related epistemological beliefs. In this socialization process, individuals are exposed to instructions about scientific explanations by the modeling explanation skills and the provision of declarative knowledge about the structure of explanations and their norms. This instruction constitutes the direct and indirect environments in the sense of Palmer and Marra’s (2008) ecological model. It should also introduce epistemological change in the sense of Bendixen’s (2002) process model of epistemological development. This process should result in an asynchronous change in the respective epistemological beliefs dimension yielding the adequate profile featured above. Individuals with such a profile should also have good explanation skills and declarative knowledge of explanation norms. However, not all individuals are necessarily exposed to this particular type of instruction. Thus, there should be profiles of epistemological beliefs that could be considered inadequate for explanations. Individuals with such a profile should also have low explanation skills and low declarative knowledge of explanation norms. The question remains whether these profiles can be demonstrated empirically.

Since any form of instruction targeted particularly at enhancing explanation skills and knowledge about explanation norms, e.g., in the form of worked examples or learning from advocatory errors, is a special form of the direct environment, it should be prone to inducing an epistemological change process. Thus, the additional question arises how an intervention changes the profiles mentioned above.

### 1.3. The person-oriented approach and its methodology

The previous section featured the idea of an epistemological beliefs profile that is adequate for scientific explanations. This idea represents a person-oriented approach. In contrast to the usual variable-oriented approach that focuses either on variables or on the relations of several variables, the person-oriented approach “is a theoretical concept at the level of phenomena, at the system level” and brings its own methodological considerations (Bergman et al., 2003, p. 23). The person-oriented approach focuses on the individual as the unit of analysis in contrast to variables. The individual is seen from a holistic and dynamic perspective. However, variables also play their role in the person-oriented approach: Individuals can be characterized by their specific pattern of the values of the variables of interest (Bergman et al., 2003, p. 24). In this way, the person-oriented approach resembles Stern’s (1900, 1994) early concept of comparative research.

The person-oriented approach focuses on the dynamic interaction of epistemological beliefs and explanation skills. Following Hickendorff et al. (2018), the person-oriented approach in learning research aims not to describe a single individual but to describe general patterns of individual characteristics and developmental pathways. These authors further state that knowing such patterns and pathways provides the foundation to understand why some learners are more successful with learning than other learners. These patterns are identified as homogeneous subgroups of individuals that show similar patterns of characteristics. Concerning patterns of change, Magnusson’s (1998) questions concerning individual change are best addressed through a person-centered approach.

The challenge in the person-centered approach from a methodological perspective is to use the appropriate methods to discover these patterns of characteristics and changes. A method to discover patterns is latent profile analysis (LPA). An LPA tries to identify classes, i.e., homogenous subgroups of individuals, that have different patterns or profiles on a set of metric observable variables (e.g., Oberski, 2016; Spurk et al., 2020). The classes represent unobservable (latent) categorial variables that give rise to the different profiles on the set of observed variables. From a statistical point of view, the different categories are represented by a multivariate mixture distribution of the pertinent observable variables.

A latent profile transition analysis is the longitudinal extension of the LPA to more than one wave of measurements. LPTA tries to identify classes with different profiles on a set of metric observable variables for all waves.^3^ LPTA can also handle situations where events, e.g., interventions, happen between measurement waves, as long as these happen to all individuals. The particularity of the LPTA consists in the simultaneous estimation of the profiles for all measurement waves at the same time, and that the number of classes can be different for each measurement wave. Additionally, an LPTA determines transition probabilities between consecutive measurement waves, i.e., the probability with which a class member changes its class membership in the following wave. Regarding the possible transitions, the interpretation has to take the temporal order into account, i.e., the only possible transitions are from the classes from a measurement wave into the classes of the following measurement wave. Thus, an LPTA enables to identify profiles of individual characteristics and their developmental path.

### 1.4. The current study

The present explorative study assesses changes in pre-teachers’ profiles of epistemological beliefs, declarative knowledge of explanation norms, and explanation skills before and after an intervention using a person-centered analysis by an LPTA. In particular, following the considerations above, we are interested in the following two research questions:

RQ1. Which profiles of epistemological beliefs, declarative knowledge about explanations, and explanation skills can be identified before and after an intervention targeted to enhance explanation skills? In particular, we are interested in

RQ1a. if there are epistemological belief profiles that go along with a high level of declarative knowledge about explanations and explanation skills before the intervention, and

RQ1b. if there are epistemological belief profiles that go along with a high level of declarative knowledge about explanation and explanation skills after the intervention.

RQ2. How do the profiles change after the intervention? In particular, which changes in patterns occur, and are there changes particularly adequate for declarative knowledge about explanation and explanation skill?

## 2. Methods

### 2.1. Sample and procedure

The participants were teacher students recruited in compulsory courses in educational psychology of a German university. The study was embedded in the regular curriculum of the course. They participated voluntarily and informed consent was obtained. The initial sample consisted of 136 participants, 28 of whom were excluded from the analysis. The exclusion resulted either from leaving the course due to various reason (e.g., a shift in their time table) or by missing one of the course sessions in which the study was conducted. Additionally, we excluded participants that left out an explanation either in the pre-ort posttest or participants that left out more than 10 % of the epistemological belief items. For the remaining participants, missing values on the epistemological belief scales were unsystematic and were therefore replaced with the mean. The final sample consists of 108 student teachers, including 67 women. The mean age was 23.04 years (*SD* = 3.84). The mean semester was 3.96 (*SD* = 2.52).

**Figure 3:**
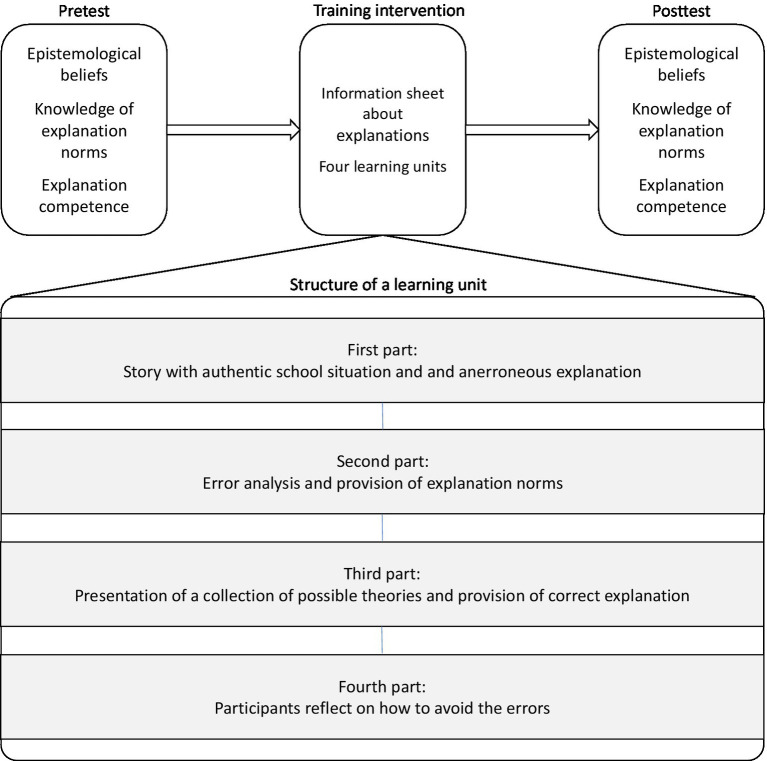
Procedure and structure of the training intervention.

All of the participants received the same treatment over four sessions. The students participated in groups, and the sessions were repeated on the same day for 4 weeks. The procedure is depicted in Figure 3 (upper part). In the first session, they took a pretest consisting of demographic questions, the epistemological belief scales, a multiple-choice questionnaire to measure declarative knowledge of explanations, and a scenario measuring explanation skills. At the end of the first session, the participants received a worksheet about the topic of scientific explanations. The worksheet was provided as homework. To ensure that the worksheet had been worked on, the edited worksheet was collected at the beginning of the next lesson. The participants worked on the first and second learning units in the second session and the third and fourth learning units in the third session. In the fourth session, the participants once more filled out the epistemological belief scales, the declarative knowledge of explanations measure, and a second scenario measuring explanation skills. In all sessions, the participants worked self-paced. The allowed maximum time was 90 min. No participant worked longer than this time frame.

### 2.2. Training intervention

The training intervention consisted of a worksheet and four paper-and-pencil learning units. Figure 3 (lower part) provides an overview of the structure of the training intervention. Before the first learning unit, the participants received a worksheet that introduced them to the concept of scientific explanations and the DN model. The worksheet contained several worked examples of explanations (cf., Klopp and Stark, 2018). Additionally, the most important explanation norms were introduced, in particular the norms pertaining to the use of current scientific theories and empirical results in contrast to everyday psychological or subjective theories, and the multiperspectivity norm.

After the worksheet, the four learning units followed. These learning units draw on the concept of learning from advocatory errors. The learning units feature a story of a teacher who explains an authentic school situation and thereby commits one or more errors, i.e., the explanation contains violations of one or more explanation norms. The character was chosen to ensure the relevance of the content for the participants and to foster the identification of the participants with the featured character. Each learning unit consisted of four parts: In the first part, the situation and the teacher’s erroneous explanation are presented to the participants. In the second part, a school psychologist, another character in the story, analyzes the teacher’s explanation and scrutinizes the errors. The participants received a multiple-choice repetition/recall test about the previously presented errors in the third part. When the participants finished the test, they received the correct answers to the questions and the instruction to compare their answers with the correct solution. Afterward, the school psychologist presented the correct explanation of the situation. Then the school psychologist outlined the actual explanation, which also entailed modeling the theory-evidence-coordination. A last element of the third part was the school psychologist’s presentation of strategies for avoiding violations of explanation norms. In the fourth and last part, the participants were instructed to reflect on how they could have avoided the violations of explanation norms featured in the teacher’s erroneous explanation. This was an open-ended question, and no solution was provided. The supplement contains a detailed description of the training intervention’s structure and contents.

### 2.3. Measures

#### 2.3.1. Epistemological beliefs

The epistemological belief measures consisted of six scales to assess Personal justification (PJ), Justification by authority (JA), Justification by multiple sources (JS), Justification by the scientific community (JC), Certainty of knowledge (CK), and Reflective nature of knowledge (RN). The PJ and JA scales were adapted from Greene et al. (2010) and Braten et al. (2014). The JS scale was adapted from Braten et al. (2014). Finally, the JC, CK, and RN scales were adapted from Moschner and Gruber (2017). The items were framed in a domain-general way. Each scale had five items that were applied in combination with a 6-point rating scale. Higher ratings correspond to a higher level in the respective beliefs, i.e., high levels represent firm beliefs. We calculated the sum score for each scale. Thus, the minimal sum score is 5, and the maximal sum score is 30 for each scale. The internal consistencies for the pre-and post-test are shown in Table 1. The items of these scales can be found in the Supplementary material.

**Table 1:** Descriptive statistics, correlations, and reliabilities of all variables.

Var.	*M*	*SD*	(1)	(2)	(3)	(4)	(5)	(6)	(7)	(8)	(9)	(10)	(11)	(12)	(13)	(14)	(15)
(1) PJ1	15.01	3.63	(0.66)														
(2) JA1	19.32	4.05	−0.17	(0.81)													
(3) JS1	25.02	3.44	0.24^*^	−0.14	(0.81)												
(4) JC1	18.02	3.93	0.18	0.16	0.36^***^	(0.62)											
(5) CK1	19.19	4.6	−0.07	0.46^***^	−0.08	0.17	(0.70)										
(6) RN1	24.92	2.47	0.02	−0.10	0.44^***^	0.13	0.02	(0.62)									
(7) KEN1	3.75	2.95	−0.06	−0.07	−0.13	0.10	−0.03	0.01	(0.89)								
(8) PES1	0.17	0.11	−0.16	−0.04	0.04	−0.14	−0.04	0.06	0.17	-							
(9) PJ2	14.31	3.70	0.66^***^	−0.08	0.19	0.07	−0.01	0.05	−0.17	−0.2^*^	(0.76)						
(10) JA2	19.34	3.68	−0.12	0.71^***^	−0.09	0.16	0.42^***^	−0.04	−0.01	0.06	−0.05	(0.79)					
(11) JS2	26.07	3.26	0.16	−0.15	0.63^***^	0.29^**^	−0.14	0.46^***^	0.02	0.11	0.10	0.01	(0.82)				
(12) JC2	18.61	4.43	0.12	0.06	0.26^**^	0.65^***^	0.03	0.17	0.15	−0.02	0.05	0.22^*^	0.52^***^	(0.75)			
(13) CK2	18.85	4.78	−0.11	0.54^***^	−0.12	0.11	0.81^***^	0.02	0.04	0.05	0.00	0.57^***^	−0.11	0.10	(0.72)		
(14) RN2	24.94	2.57	0.06	−0.09	0.5^***^	0.17	−0.10	0.64^***^	0.09	0.16	0.06	0.11	0.64^***^	0.32^**^	0.00	(0.75)	
(15) KEN2	10.52	1.60	−0.19^*^	−0.13	0.02	0.01	−0.17	−0.13	0.17	0.25^*^	−0.13	−0.08	−0.05	−0.09	−0.22^*^	−0.12	(0.73)
(16) PES2	0.22	0.10	0.03	−0.01	0.09	0.00	−0.14	−0.05	0.05	0.16	0.00	0.05	0.08	0.04	−0.01	0.07	0.35^***^

PJ, personal justification; JA, justification by authority; JS, justification my multiple sources; JC, justification by the scientific community; CK, certainty of knowledge; RK, reflective nature of knowledge; KEN, declarative knowledge of explanations; PES, percentage explanation score. The numbers behind the abbreviations indicate the pretest (1) and posttest (2).

* *p* < 0.05, ** *p* < 0.01, *** *p* < 0.001.

#### 2.3.2. Declarative knowledge about explanation norms

We applied a multiple-choice assessment to measure declarative knowledge of explanations (KEN). The test was adapted from Klopp and Stark (2018) and consisted of 12 questions that asked for the norms a valid explanation should follow. In particular, the test asked for six explanation norms. Additionally, the test asked for the following combination of two norms: the circular argument in combination with fundamental attribution error, the use of an inadequate theory in combination with a subjective one, the superficial interpretation of outdated empirical results, and the premature closure in combination with a one-sided viewpoint in explanations. Each item had three answer options, one of which was the attractor, while the other two were distractors. The participants were instructed not to guess and leave a blank if they did not know the correct answer. Each correct answer was awarded 1 point, and incorrect or missing answers were awarded 0 points. The points were added. Thus, the minimum number of points was 0, and the maximum number of points was 12. The split-half-reliabilities for the pre-and post-test are shown in Table 1.

#### 2.3.3. Explanation skills

We assessed explanation skills using a scenario-based test. The scenario consisted of an authentic school situation, and the participants were asked to provide a written explanation using scientific theories. In addition to the scenario, the participants received a collection of theories and were asked to use them for their explanations. The written explanations were coded for several criteria reflecting the norms for a valid explanation. Following Klopp and Stark (2018), a valid explanation should explicitly mention the theory used in the explanation. The theory has to fit the situation. There should also be a logically correct deduction from the explanans to the explanandum and an elaboration of the theory-evidence-coordination. Therefore, the written explanations were rated on whether they mentioned the theory (Theory mentioned). Points were assigned according to the level of detail the participants provided about the theory. We rated if the written explanations showed Theory-evidence-coordination, i.e., the abstract elements are matched to the pertinent elements of the situation for both the explanans and the explanandum. Please note that the rating of Theory-evidence coordination is independent of the rating in the Theory mention category, i.e., if a participant does not explicitly mention the theory but the answer is consistent with the theory and shows a fit with the available evidence, the points for the category Theory-evidence-coordination are assigned in accordance with the rating criteria. The norm of Logical correctness depends on a correct theory-evidence-coordination. We rated if the relation of the explanandum was logically deduced from the explanans. To fulfill the norm of Multiperspectivity, an explanation must consider possible alternative explanations using other possible theories that may fit the situation and mention why this alternative was not used in the first place. Additionally, the explanation should exclude theories that do not fit the situation well. The supplement contains a description of the scenarios and the coding criteria.

The rating procedure was as follows: We rated the degree to which an explanation met the above-mentioned norms and awarded points accordingly. The points were summed to obtain an overall measure of explanation skills, i.e., the explanation score. The maximum number of points for ES in the pretest was 22, and the maximum in the post-test was 33. Because the pre-and the post-test had a different number of maximum points, the ES was converted to a percentage score (PES, percentage explanation score). The rating procedure was aligned with the one in Klopp and Stark (2018): The first author set up a sample solution for the explanations in the pre-and post-test. Afterward, two independent raters, i.e., the first author and a research assistant holding a bachelor’s degree in psychology, coded nine randomly drawn explanations from the pre-and post-tests without knowledge on whether they stemmed from either the pre-or the post-test. The research assistant was not involved in the study, and both raters had experience with written explanations in rating. In the first round, both raters rated three explanations from the pretest and three explanations from the post-test according to the scheme provided in the supplement. We recorded the number of congruent ratings, which was 85% in the first round. Incongruent ratings were discussed and resolved. In some cases, a third researcher with experience in rating explanations was consulted. In the second round, another three explanations, each from the pre-and post-test were rated, and the numbers of congruent ratings were recorded. There was an agreement in 94% of the ratings, and non-agreements were resolved by discussion. In the third round, both raters rated the remaining three explanations from the pre-and the post-test. In this round, there was an agreement in again 94% of the ratings and the research assistant rated the remaining explanation without knowledge on whether they stemmed from the pre-or the post-test.

### 2.4. Statistical analysis

To carry out a person-centered analysis of the epistemological belief scales together with the KEN and PES scales, we used a Latent profile transition analysis (LPTA). The LPTA was carried out using Mplus (Muthén and Muthén, 1998-2017, version 8.8) and the R (R Core Team, 2020, version 4.0.2) with MplusAutomation package (Hallquist and Wiley, 2018, version 1.1.0). The basic LPTA model in this study consists of means and variances of the epistemological beliefs, KEN, and PES measures for which classes were specified for the pretest and post-test measurements. The classes were allowed to differ in their variances, and the residuals of the corresponding scales were allowed to covary between the two measurement occasions. The residual covariations were set equal for all classes. The first step in the LPTA is to decide on the number of classes for each of the two measurement occasions. We modeled different class numbers for both measurement occasions to achieve this goal. Due to model identification reasons, the maximum number of classes for each measurement occasion was two, so there were four possible solutions: 1) one class for the first and one class for the second measurement occasion, 2) two classes for the first and one class for the second measurement occasion, 3) one class for the first and two classes for the second measurement occasion, and 4) two classes for the first and the second measurement occasion. We estimated each of these models with 1,000 different sets of starting values in the initial stage, and the number of optimizations was set to 100 in the final stage. The maximal number of iterations in the initial set was set to 100. We implemented these settings with the STARTS and SITERATIONS options. Finally, we specified the numerical integration by using the ALGORITHM = INTEGRATION option.

To compare the four models, we used the AIC, BIC, and aBIC. These should be at their minimum at the optimal solution. We also used the density plots of the involved variables to determine the number of classes. As a final statistical criterion, we looked at the entropy, a standardized measure indicating the classification accuracy for a given model. This should be reasonably high, at best values over or equal to 0.80 (Celeux and Soromenho, 1996). In addition to these statistical criteria, we had the requirement that the final solutions must be interpretable.

To check the stability, i.e., to avoid local optima of the selected solution, we doubled the number of starting values, optimizations, and iterations in the initial set. The best likelihood from the prior analysis should be repeated. For all further analysis, we noted the seed of the best likelihood and used the OPTSEED option of Mplus.

We used the means of the variables in each cluster to interpret the clusters. If necessary, we also considered the univariate entropies, i.e., a standardized measure indication that tells us how much a given variable contributes to the separation of classes. The univariate entropies are compared to get the variables over one class to provide a rank order of how they separate. There is no agreed minimal threshold for this measure. To enrich the person-centered interpretation of the classes with a variable-oriented perspective, we compared the variables between the classes. In particular, we first compared the variables between the classes in the pre-test and the post-test using a Wald test. In this case, the Wald test provides a *χ*^2^-distributed test statistic with one degree of freedom. In Mplus, we implemented the Wald test using the MODEL TEST command. We used Cohen’s *d* (Cohen, 1988) to measure effect size for class comparisons.

Finally, we evaluated the class transitions between the measurement times expressed by the transition probabilities between the classes. Again, to enhance this person-centered perspective with a variable-oriented view, we compared the means of the variables for each of the possible transitions. To get the difference, we defined a parameter representing the difference of the post-test value of a variable minus its pretest values. A significance test is then obtained using the delta method, which provides the defined parameter’s standard error. In Mplus, we implemented this procedure using the MODEL CONSTRAINT command. Again, we used Cohen’s *d* as a measure of effect size.

For the remaining analyses, we used the packages psych (Revelle, 2020, version 2.0.9) for the psychometric analysis, psytabs (Beller, 2016, version 1.0) for the display of the results as well as ggplot2 (Wickham, 2016, version 3.3.2) to create the density plots and package the patchwork (Pedersen, 2020, version 1.1.1) to arrange the plots.

## 3. Results

### 3.1. Descriptive analysis

**Figure 4:**
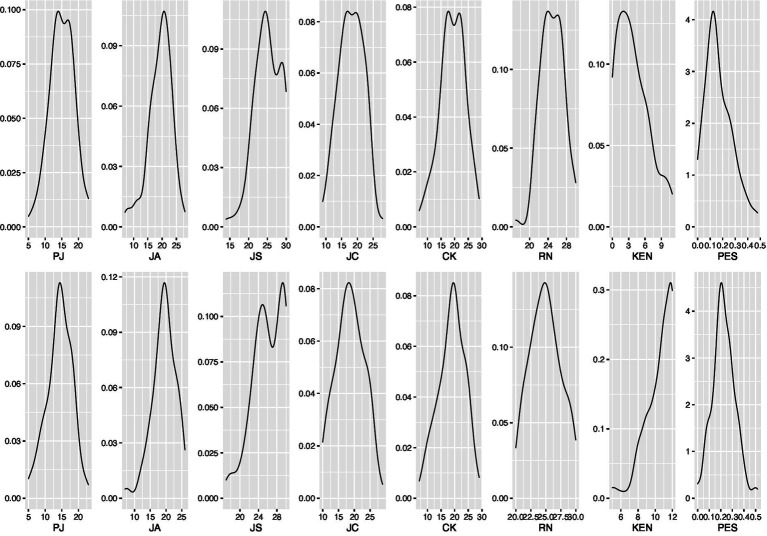
Density plot for all variables, the first row contains the pretest and the second row the post-test variables. PJ, personal justification; JA, justification by authority; JS, justification my multiple sources; JC, justification by the scientific community; CK, certainty of knowledge; RK, reflective nature of knowledge; KEN, declarative knowledge of explanations; PES, percentage explanation score.

The descriptive statistics for all variables in the analysis are shown in Table 1, and Figure 4 shows the density plots. Concerning the overall changes in epistemological beliefs, KEN, and PES, we conducted pairwise *t*-tests for each variable. There is a significant decline in PJ, *t*(106) = −2.309, *p* = 0.023, a significant increase in JS, *t*(106) = 3.824, *p* < 0.001, as well as a significant increase in KEN, *t*(106) = 22.499, *p* < 0.001, and PES, *t*(106) = 4.334, *p* < 0.001. Table 1 also shows almost no overall change for JA and RN. For JC, there is a descriptive increase, whereas, for CK, there is a descriptive decrease. Epistemological beliefs show a change pattern consistent with our theoretical expectations. Furthermore, Table 1 shows that the epistemological belief measures in the pretest correlate significantly with the epistemological belief measures in the post-test, justifying the modeling of the residual covariances between these variables. The correlational analysis also reveals that there is neither a significant correlation between pretest epistemological beliefs and PES nor a significant correlation between pretest epistemological beliefs and KEN. However, there is a significant negative correlation between CK and KEN for the post-test measures.

Concerning the distributional form, the variables in the pretest are shown in the upper row of Figure 4. There are bimodal distributions for PJ, JS, JC, CK, and RN. Additionally, KEN and PES are positively skewed, indicating that knowledge of explanation norms and explanation skills is rather low. The distributional forms changed considerably after the training intervention. The density plots are shown in the lower row of Figure 4. Concerning the epistemological beliefs scales, only the JS scale shows a clear bimodal form. The distributions for KEN and PES are now negatively skewed, indicating an increase in knowledge of explanation norms and skills. Additionally, the overall increase in KEN and PES shows that learning from advocatory errors indeed fosters the student’s skills in constructing explanations.

### 3.2. Latent profiles in the pre-and post-test (RQ1)

The first step in an LPTA consists in determining the optimal number of classes to be chosen. Table 2 shows the AIC, BIC, aBIC, and the entropies for the various solutions. The AIC and the aBIC indicate a 2-class solution in both the pretest and the post-test, whereas BIC points to a 1-class solution in the pretest and a 2-class solution in the post-test. Considering the density plots, we followed the AIC and aBIC and opted for the 2-class solution in both the pretest and the post-test. These classes are indicated as follows: The classes in the pretest are referred to as C1, with C1.1 being the first class and C1.2 being the second class. The classes in the pretest are referred to as C2, with C2.1 being the first class and C2.2 being the second class. The entropy of the final solution is 0.800, and the class-specific entropies are 0.852 for C1 and 0.712 for C2. For this solution, the estimation process replicated the best likelihood. To check the 2-class solution, we duplicated the number of replications. The best likelihood was replicated again. Thus, we consider the results stable.

**Table 2:** Information criteria and entropies.

Number of classes^*^	AIC	BIC	aBIC	Entropy
1/1	7239.345	7346.630	7220.242	-
1/2	7203.266	7356.147	7176.044	0.787
2/1	7214.341	7367.222	7187.119	0.865
2/2	7156.832	7357.991	7121.014	0.800

* Number of classes for the pretest/Number of classes for the post-test.

Table 3 (left panel) shows the mean profiles, the variances, and the univariate entropies for the classes C1.1 and C1.2 resulting from the pretest. The table shows that the classes differ considerably in the variables’ variances. This result justifies our decision to avoid equality constraints on the variances in the model. Table 4 shows the Wald tests comparing the means between the classes C1.1 and C1.2. In the pretest, the two classes do not differ in KEN, but class C1.1 (*N* = 81) has a significantly higher PES than class C1.2 (*N* = 27). This difference also has a large effect size. The two classes differ significantly in JA, JS, and RN. The first class has a significantly lower JA level with a medium effect size for the difference. At the same time, class C1.1 had significantly higher levels in JS and RN, with large effect sizes for both differences. Thus, class C1.1 contains those individuals with epistemological beliefs adequate for making explanations. Table 3 (right panel) shows the mean profiles, the variances, and the univariate entropies for the classes C2.1 and C2.2 resulting from the post-test, and Table 5 shows the Wald tests comparing the means between these classes. In the post-test, the classes C2.1 and C2.2 only differ significantly in KEN and PES, both differences showing large effect sizes, but there are no differences in epistemological beliefs. Class C2.1 (*N* = 61) has smaller KEN and PES levels than class C2.2 (*N* = 47).

**Table 3:** Means, variances, and univariate entropies for all classes.

	Pretest C1*E* = 0.854					Post-test C2*E* = 0.712				
	C1.1		C1.2			C2.1		C2.2		
	*N* = 81		*N* = 27			*N* = 61		*N* = 47		
	*M*	*S^2^*	*M*	*S^2^*	*H*	*M*	*S^2^*	*M*	*S^2^*	*H*
PJ	15.103	16.633	14.721	10.013	0.124	13.958	15.508	14.785	8.030	0.147
JA	19.013	16.549	20.286	10.998	0.132	19.288	15.341	19.418	12.025	0.124
JS	25.456	10.751	23.667	9.867	0.142	26.431	10.569	25.583	9.897	0.126
JC	17.890	20.325	18.417	10.627	0.127	18.982	17.366	18.100	21.091	0.125
CK	19.194	22.684	19.157	15.328	0.125	19.052	25.188	18.576	20.766	0.122
RN	25.344	6.821	23.594	3.666	0.164	24.989	7.657	24.861	5.109	0.126
KEN	3.757	8.133	3.730	10.003	0.122	9.671	2.459	11.687	0.229	0.376
PES	0.195	0.012	0.084	0.002	0.222	0.201	0.009	0.259	0.008	0.155

Designation for classes: C1 – classes at pretest, C2 – classes at posttest. Class names are: C1.1 – skilled explainers with adequate epistemological beliefs, C1.2 – unskilled explainers with inadequate epistemological beliefs, C2.1 – skilled explainers, C2.2 very skilled explainers. H, univariate entropy; E, class specific entropy; PJ, personal justification; JA, justification by authority; JS, justification my multiple sources; JC, justification by the scientific community; CK, certainty of knowledge, RK, reflective nature of knowledge; KEN, declarative knowledge of explanations; PES, percentage explanation score.

**Table 4:** Wald tests for comparing the two classes at pretest and effect sizes (Cohen’s *d*).

Variable	Class C1.1	Class C1.2	*χ* ^2^	*p*	*d*
PJ	15.103	14.721	0.655	0.418	0.669
JA	19.013	20.286	6.320	0.012	0.324
JS	25.456	23.667	6.883	0.009	0.546
JC	17.890	18.417	0.745	0.388	0.123
CK	19.194	19.157	0.007	0.931	0.008
RN	25.344	23.594	27.676	<0.001	0.705
KEN	3.757	3.730	0.001	0.975	0.009
PES	0.195	0.084	49.259	<0.001	1.125

df = 1 for each Wald test. PJ, personal justification; JA, justification by authority; JS, justification my multiple sources; JC, justification by the scientific community; CK, certainty of knowledge; RK, reflective nature of knowledge; KEN, declarative knowledge of explanations; PES, percentage explanation score.

**Table 5:** Wald tests for comparing the two classes at post-test and effect sizes (Cohen’s *d*).

Variable	Class C2.1	Class C2.2	*χ* ^2^	*p*	*d*
PJ	13.958	14.785	2.337	0.126	0.222
JA	19.288	19.418	0.023	0.880	0.034
JS	26.431	25.583	0.965	0.326	0.260
JC	18.982	18.100	0.683	0.424	0.204
CK	19.052	18.576	0.564	0.453	0.096
RN	24.989	24.861	0.067	0.795	0.048
KEN	9.671	11.687	69.053	<0.001	1.444
PES	0.201	0.259	7.181	0.007	0.614

df = 1 for each Wald test. PJ, personal justification; JA, justification by authority; JS, justification my multiple sources; JC, justification by the scientific community; CK, certainty of knowledge; RK, reflective nature of knowledge; KEN, declarative knowledge of explanations; PES, percentage explanation score.

Concerning RQ1a, the results for the pretest are in line with our theoretical expectations about the adequate aspects of epistemological beliefs on the endeavor of scientific explanation. In particular, the higher value of JS indicates that the appreciation of multiple sources for scientific claims goes along with better explanation skills, perhaps due to the requirement to consider alternative theories in an explanation. Similarly, those individuals with higher levels of RN have higher explanation skills. This may be because of a tendency to critical questioning which is also adequate to set up a valid explanation. This tendency may also reflect a better approach to learning about scientific explanations, either explicitly or implicitly, by revising the naïve conceptions of an explanation that go along with low explanation skills. However, there was no difference in the knowledge of explanation norms, and in particular, the individuals in both classes had rather low levels of KEN. Concerning the class separations, the univariate entropies shows that PES contributes most to the class separation, followed by RN, JS, JA, JC, CK, PJ, and KEN. Therefore, we interpret class C1.1 as *skilled explainers with adequate epistemological beliefs*. In contrast, we interpret class C.1.2 as *unskilled explainers with inadequate epistemological beliefs*.

Concerning RQ1b, the training intervention seems to equalize the epistemological profiles for the two classes in the post-test (see Table 5). At least, there are no significant differences between the epistemological belief profiles indicating any particular epistemological belief in one class that is adequate for explanation compared to the other class. However, there are significant differences between KEN and PES. The univariate entropies show that KEN is the variable that separates the classes most, followed by PES, PJ, JS and RN with a tied rank, JC, JA, and CK. Thus, these two classes differ mostly with respect to the variables relating to explanations. Because of the absence of marked differences between epistemological beliefs, we interpret class C2.1 as *skilled explainers* and class C2.2 as *very skilled explainers*.

### 3.3. Transitions between pretest and post-test categories (RQ2)

**Figure 5:**
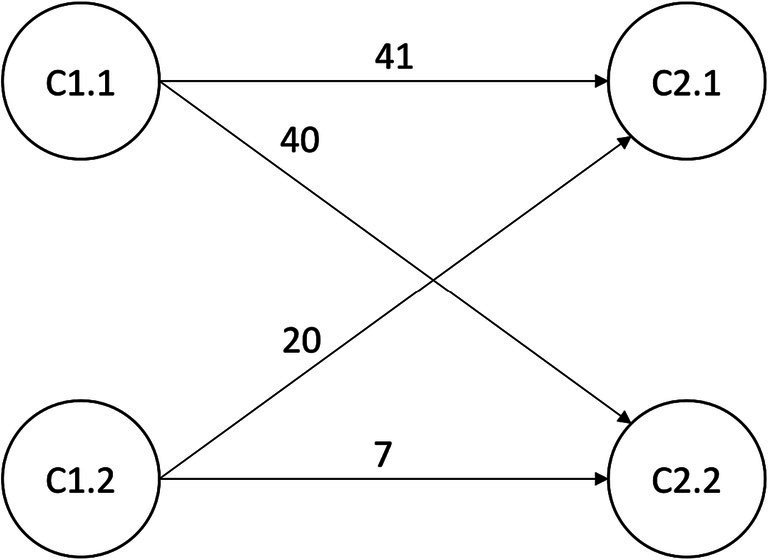
Pathway plot of the class transition between pre-and posttest.

Concerning these changes, i.e., the transition from one class in the pretest to another class in the post-test, Table 6 shows the transition probabilities and the number of individuals in each transition category, see also Figure 5 for a pathway plot. For ease of reference, we denote the cells in Table 6 as transition categories and then from one to four, as indicated in the table. For each of these transition categories, Table 7 provides the differences, i.e., the changes in epistemological beliefs, KEN, and PES. In the first transition category, i.e., the transition from class C1.1 (*skilled explainers with adequate epistemological beliefs*) to class C2.1 (*skilled explainers*), there are *N* = 41 individuals. Table 7 shows that in this transition category, there is a significant decline in PJ with a medium effect size and a significant increase in KEN with a large effect. In the second transition category, i.e., the transition from class C1.1 (*skilled explainers with adequate epistemological beliefs*) to class C2.2 (*very skilled explainers*), there are *N* = 40 individuals. In this category, there are no significant changes in epistemological beliefs but significant increases in KEN and PES, both with large effect sizes. In the third transition category, i.e., the transition from class C1.2 (*unskilled explainers with inadequate epistemological beliefs*) to class C2.1 (*skilled explainers*), there are *N* = 20 individuals. There is a significant decrease in JA with a medium effect and significant increases in JS and RN, both showing large effect sizes. Additionally, there are significant and large increases in KEN and PES. In the fourth and last transition category, i.e., the transition from class C1.2 (*unskilled explainers with inadequate epistemological beliefs*) to class C2.2 (*very skilled explainers*), there are N = 7 individuals. In this transition category, there is a significant decrease in CK with a small effect size and significant and large increases in JS and RN. There are significant and large increases in KEN and PES.

**Table 6:** Transition probabilities and transition frequencies.

Classes		Post-test	
		C2.1	C2.2
Pretest	C1.1	0.529/41 (1)	0.471/40 (2)
	C1.2	0.737/20 (3)	0.263/7 (4)

The numbers in brackets indicate the numbering of the transition categories as mentioned in the text. Class names are: C1.1 – skilled explainers with adequate epistemological beliefs, C1.2 – unskilled explainers with inadequate epistemological beliefs, C2.1 – skilled explainers, C2.2 very skilled explainers.

**Table 7:** Differences between the profiles in class changes and effect sizes (Cohen’s *d*).

Variable	Difference	*SE*	*p*	*d*
Change from class C1.1 to class C2.1				
PJ	−1.143	0.442	0.010	0.283
JA	0.278	0.505	0.582	0.068
JS	0.984	0.540	0.068	0.296
JC	1.098	0.678	0.105	0.248
CK	−0.140	0.425	0.742	0.029
RN	−0.350	−0.350	0.316	0.132
KEN	5.916	0.386	<0.001	2.459
PES	0.005	0.019	0.774	0.058
Change from class C1.1 to class C2.2				
PJ	−0.319	0.451	0.479	0.086
JA	0.396	0.537	0.461	0.104
JS	0.119	0.557	0.831	0.039
JC	0.199	0.729	0.784	0.046
CK	−0.625	0.543	0.250	0.131
RN	−0.491	0.377	0.194	0.192
KEN	7.932	0.337	<0.001	3.434
PES	0.064	0.018	0.001	0.618
Change from class C1.2 to class C2.1				
PJ	−0.759	0.559	0.174	0.203
JA	−0.993	0.504	0.049	0.263
JS	2.770	0.591	<0.001	0.849
JC	0.571	0.433	0.187	0.143
CK	−0.097	0.599	0.871	0.022
RN	1.406	0.495	0.005	0.543
KEN	5.942	0.698	<0.001	2.697
PES	0.116	0.015	<0.001	1.394
Change from class C1.2 to class C2.2				
PJ	0.064	0.064	0.315	0.021
JA	−0.875	0.628	0.164	0.251
JS	1.904	0.837	0.023	0.601
JC	−0.327	0.947	0.730	0.075
CK	−0.582	0.168	0.001	0.132
RN	1.265	0.082	<0.001	0.583
KEN	7.959	0.725	<0.001	4.047
PES	0.175	0.016	<0.001	2.259

PJ, personal justification; JA, justification by authority; JS, justification my multiple sources; JC, justification by the scientific community; CK, certainty of knowledge; RK, reflective nature of knowledge; KEN, declarative knowledge of explanations; PES, percentage explanation score.

In the transition from *skilled explainers with adequate epistemological beliefs* to *skilled explainers* (first transition category), we see that there is only a decline in PJ, see Table 7. This may be because these individuals already had adequate epistemological beliefs in their starting class. In this transition category, there is lack of a significant increase in PES. However, there is a significant increase in KEN. In the transition from *skilled explainers with adequate epistemological beliefs* to *very skilled explainers* (second transition category), we see no essential change in epistemological beliefs. This may be because these individuals already had adequate epistemological beliefs in their starting class. However, there is a significant increase in both KEN and PES. The characteristics of the changes in the transition differ strongly from the third and fourth transition category. Whereas there are only slight changes in epistemological beliefs in the first two transition categories, there are marked changes in epistemological beliefs in the third and fourth transition category. In the transition from *unskilled explainers with inadequate epistemological beliefs* to *skilled explainers* (third transition category), the pattern of changes in epistemological beliefs corresponds to the theoretical expectation of a change toward adequate epistemological beliefs. The same holds in the transition from *unskilled explainers with inadequate epistemological beliefs* to *very skilled explainers* (fourth transition category). But there are differences in the epistemological belief changes in both transition categories. However, marked increases in KEN and PES exist in both categories. The transition categories’ frequencies are an additional observation. Whereas most participants are in the first and second transition category, fewer participants are in the third and fourth transition categories. However, the rank order according to the transition probabilities is third, first, second, and fourth transition category, see Table 6. Thus, the most likely transition is from *unskilled explainers with inadequate epistemological beliefs* to *skilled explainers*, but only approximately one-fifth of the participants belong to this category. The transition from *skilled explainers with adequate epistemological belief* to either *skilled explainers* or *very skilled explainers*, respectively, is about equally likely. Also, the majority of the participants belong to these two transition categories. The least likely transition to which only a minority of the participants belongs is the transition from the *unskilled explainers with inadequate epistemological beliefs* to the *very skilled explainers*. This last result is not very surprising. These are the learners with the worst prerequisites, so a high learning gain would only be expected in very few cases.

## 4. Discussion

The results of the LPTA are generally in accordance with the theoretical reasoning about epistemological beliefs and their relation with the explanation skills and knowledge about explanations. Concerning RQ1a, the results indicated two different profiles before the intervention: The skilled explainers with adequate epistemological beliefs had significantly better explanation skills than unskilled explainers with inadequate epistemological beliefs but did not differ in their declarative knowledge about explanations, which is an unexpected result. Intuitively, the expectation is that individuals with higher levels of declarative knowledge about explanations should have better explanation skills. Given the mutual development of epistemological beliefs, explanation skills, and declarative knowledge about explanations during the enculturation in the domain (Palmer and Marra, 2008; Klopp and Stark, 2016), this result seems to imply that epistemological beliefs are primarily important for the development of explanation skills but not for the declarative knowledge about the structure and norms of explanations. The skilled explainers may apply the standards resulting from their epistemological beliefs to explanations. However, from a developmental perspective, we could not ascertain whether the development of adequate epistemological beliefs yields better explanation skills, or if the exposition to scientific content like explanations yields an adequate profile of epistemological beliefs. In the second case, acquiring explanation skills may yield an adequate epistemological belief profile. Moreover, these results reveal that the importance of epistemological beliefs is only visible from a person-oriented perspective, as evidenced by the lacking correlations between explanation skills, declarative knowledge, and epistemological beliefs.

However, for the pretest, there were only significant differences in Justification by authority, which is lower for the skilled explainers with adequate epistemological beliefs, and for Justification by multiple sources and Reflective nature of knowledge, which both are higher for the skilled explainers with adequate epistemological beliefs. These results align with the theoretical reasoning about the relations between epistemological beliefs and explanations. There may be different reasons for the absence of significant differences for the other three epistemological belief dimensions. For example, for Personal justification, the overall scale value is quite low in comparison to the theoretical maximum of the scale and also in comparison with the other epistemological belief measures. Considering that most of the participants are in their third and fourth semester (see section 2.1), the overall level of Personal justification might have declined during their course of studies in such a way that class differences are no longer relevant for explanation skills. This decline might be a result of the exposure to scientific content and methods. However, such an interpretation bases on additional information about the general level of and the development of Personal justification in the student population. For Certainty of knowledge, similar reasoning as for Personal justification may apply even though the class means for these two dimensions are higher. However, for Justification by the scientific community, the reasons for the missing differences between the classes at the pretest may differ. In Germany, teacher education focuses on evidence-based scientific reasoning, which includes the view that scientific knowledge rests on empirical findings, at best corroborated in multiple studies. In turn, this should lead to a decrease in the belief in Justification in authority and an increase in the belief in Justification by multiple sources. However, the concept of a scientific community is neither explicitly taught nor are the students in this early stage of academic development implicitly introduced to this concept. Thus, potential differences in Justification by the scientific community could not arise and not relate to differences in explanation skills. However, the absence of significant class differences may also have a statistical reason related to the large variances of the epistemological belief measures within these classes. These large variances, in turn, yield large standard errors resulting in non-significant parameters such that the class differences are too small to differ significantly.

Regarding RQ1b, we firstly observe that the training intervention seems to level out the effects of epistemological beliefs on explanation skills and declarative knowledge about explanations, as evidenced by the lack of significant differences in epistemological beliefs between the post-test profiles. A possible reason is that the intervention affects epistemological beliefs so that an “optimum” adequate profile emerges. However, this does not mean that epistemological beliefs are unimportant after the intervention. It is possible that acquired explanation skills and knowledge about explanations are dominant due to the intervention shortly before and temporarily overshadow the effects of epistemological beliefs. Additionally, differences between the profiles may not result in significant differences due to large variances and/or a lack of statistical power.

Concerning the differences between the profiles, it should be noted that comparisons between the classes are only relative. As there are no reference at which level a sum score represents a low or high level, the profile of a class cannot be judged on its own but only in comparison with the other class. Thus, for the “leveling out” of the profiles, there cannot be an answer if the profiles reflect high, low or “optimal” beliefs level. The only thing that can be judged is the level change, which we consider in the second research question.

Concerning RQ2, we consider the transitions between the classes starting with the first and second transition category. For the change from the skilled explainers with adequate epistemological to the skilled explainers (first transition category), there is a decrease in Personal justification and an increase in declarative knowledge about explanations. Concerning explanation skills, there is only a slight increase. For the change from the skilled explainers with adequate epistemological to the skilled explainers (second transition category), there are only sharp increases in explanation skills and declarative knowledge about explanations. The change in the belief in Personal justification only in the first transition category seems unexpected at first glance. Still, it is in line with the theoretical expectation that a high level of Personal justification is inadequate for explanation skills. As there is no improvement in explanation skills, the decline in Personal justification is likely related to the increase in declarative knowledge about explanations. Learning that explanations have a certain structure and are based on norms may diminish the respective belief that scientific assertions like explanations are merely the personal opinions of scientists.

Regarding the third transition category, we have the transition from the unskilled explainers with inadequate epistemological beliefs to the skilled explainers in the third transition category. The change of Personal justification is in accordance with theoretical expectations as the decline corresponds to a change towards an adequate epistemological belief. The same pattern of changes towards more adequate epistemological beliefs emerged in the third and fourth transition category. A notable result is the increase in the Reflective nature of knowledge in both transition categories. In the sense of Bendixen’s (2002) process model, the exposition to the scientific explanation concept and the learner’s need to change from a naïve to a scientific explanation concept may have made obvious the incongruence of a weak belief in the Reflective nature of knowledge. This, in turn, may have finally triggered epistemological doubt resulting in the observed epistemological belief change. In contrast to the third transition category, there is no significant change in Justification by authority in the fourth category. This change is close to the significance level in the third category, and thus the smaller change in the fourth category was not large enough to become statistically significant, as indicated by the slightly lower effect size.

Additionally, in the fourth transition category, there is also a decrease in the belief in the Certainty of knowledge which is in accordance with theoretical reasoning, and the same considerations as for the Reflective nature of knowledge apply. However, we have yet to determine why this change only occurs in the fourth transition category. Possibly, as this category contains the transition from the unskilled explainers with inadequate epistemological beliefs to the very skilled explainers, there may have been more issues that were incongruent with the current belief resulting in the decline in Certainty of knowledge. In addition, the marked changes in the Reflective nature of knowledge, i.e., an epistemological belief that directly relates to learning (cf., Moschner and Gruber, 2017), in combination with large and significant differences between the classes at the pretest measurement, the results in the fourth transition category show that a learning process can affect epistemological beliefs in a direction that is itself adequate for learning. So, the learners that profited most in terms of their explanation skills and declarative knowledge about explanations also experienced the most profitable change in epistemological beliefs. Similar considerations apply in the third transition category, but the increase is smaller than in the fourth transition category.

## 5. Limitations

The present study has several limitations. Firstly, there are methodological limitations. We already mentioned the limitation of the number of classes that could be identified using the LPTA. The number of parameters of interest, i.e., the class-specific means and variances that can be identified, is determined by the number of observed variables and the number of classes. For a given number of class-specific parameters and observed variables, only a limited number of classes can be specified in the model. In the current setting, the number of classes was two for both measurement occasions. If more classes had to be estimated, then the number of estimated parameters per class would have had to be restricted, for instance, by setting the variances equal across parameters, which is a standard assumption in an LPA. However, as seen, the estimated variances per class are largely different such that this restriction would have biased the other estimated parameters. Additionally, the plots of the observed variables did not indicate more than two classes at any measurement wave due to the absence of visually recognizable trimodal distributions. From a methodological perspective, there is also the question of how to include the variables declarative knowledge about explanations and explanation skills in the model. In the current study, these variables were added to the variables used in the class determination. This was done to account for the interplay of epistemological beliefs and explanation skill variables in accordance with the person-centered approach. Technically, however, these two variables could also have been modeled as dependent variables. In this case, the latent classes would have been determined based on the epistemological beliefs only, and the classes would have been considered as the causes for explanation skills and knowledge about explanations.

A second limitation is the duration of the training intervention. From the pre-test to the post-test, the total duration was 4 weeks. Thus, the effects of the training intervention may not be separated from the effects stemming from the participant’s academic environment. However, at least for the domain of educational sciences, the standard curriculum for the participants shows that the topic of scientific explanation was not covered during the time of the training intervention. Additionally, the large time frame and the week between the last training session and the post-test may have neutralized some change in epistemological beliefs so that they did not manifest in the pattern of changes in the class transitions.

A third limitation results from the explanations skills test. These tests were different for the pre-and post-test. Although the tests were normalized by calculating the percentages of the scores, the different tests may have had different requirements, in particular regarding the domain-specific prior knowledge from educational psychology. However, as the necessary theory was provided in both tests, the effects of different domain-specific prior knowledge about the respective contents should be neglectable.

Fourthly, other epistemological beliefs are potentially relevant to constructing explanations. For example, Barzilai and Weinstock (2015) identify the dimension Evaluation of explanations, Reliable explanation, and Multiple perspectives. Evaluation of explanation describes interindividual differences in the belief that valid explanations should rely on data, not opinions. Reliable explanation describes interindividual differences in the belief that a scientific explanation should draw on a theory rather than personal knowledge or opinions. Finally, multiple perspectives describe interindividual differences in the belief that considering more than one position contributes to a balanced way of thinking. Thus, these dimensions would be of direct relevance. However, the measurement scales developed by Barzilai and Weinstock (2015) are designed to capture epistemological beliefs in the sense of the three developmental levels. They cannot be easily transformed to measure interindividual differences in the sense of the dimensional approach.

The final limitation refers to the alignment between the various components of explanation skills and epistemological beliefs. In this study, we operationalized explanation skills as the sum score of its components. However, there are certain dimensions of epistemological beliefs that are more closely related to certain components of explanation skills than to others. For instance, epistemological beliefs like the justification by multiple sources may be more closely linked to the component multiperspectivity than to other components. In the same vein, the beliefs in personal justification and justification by authority which are related to the choice of a specific theory for the construction of an explanation, may match the component theory-evidence-coordination closer than any other components. Thus, an analysis on the level of the components of explanation skills might yield a more fine-grained picture of the relation between epistemological beliefs and the determining factors in the construction of an explanation. At the same time, considering the distinct components of explanation skills on their own would increase the number of observed variables so that more classes could be taken into the model (see the first limitation).

## 6. Implications for research

This study has shown that a person-centered approach is a viable perspective on epistemological beliefs and extends other work in this direction (e.g., Kampa et al., 2016; Hickendorff et al., 2018). The study once more demonstrated the viability of a person-centered approach to study epistemological beliefs by demonstrating that it is possible to identify homogenous subgroups of individuals and to describe the individuals using meaningful and theoretically consistent profiles. Additionally, the vast absence of correlation between epistemological beliefs and performance measures at both measurements indicates that a person-centered approach allows insights hidden in a variable-centered approach.

From a methodological perspective, it would be worthwhile for future studies to focus not only on either a variable-or person-centered approach. A combination of both approaches would allow insights into different profiles of epistemological beliefs and the relations of the various variables with the different profile classes. The results would be qualitatively different classes in which the relations between the variables may be quantitatively analyzed. Additionally, a quantitative analysis to examine the differences between the profiles could be applied, as shown in the current study. From a statistical point of view, such an approach would entail the use of mixture models, e.g., mixture regression models.

The integrated approach also benefits the study of epistemological belief change. Most of the newer studies on epistemological belief interventions (e.g., Rosman et al., 2016; Klopp and Stark, 2022a,b) draw on the notion of the three levels of epistemological development. However, this is a rather crude change model. As the integrated approach describes these levels in terms of profiles, a person-centered one seems to be a natural application of the integrated approach. In addition, and as already mentioned above, a variable-centered analysis could be carried out within the identified subgroups.

A new element in this study was the use of an LPTA to study the effects of an intervention on epistemological beliefs. Typically, LPTA is used to analyze naturally occurring change processes. In studies on experimentally induced epistemological belief change (e.g., Rosman et al., 2016; Klopp and Stark, 2022a,b), a measure of change is used that is analyzed with the statistical methods to compare the change measure between several groups, e.g., between a control group and one or more experimental groups. Although there was only one group in this study, the approach of the LPTA could potentially be used to determine epistemological belief profiles beforehand and then apply specific interventions to these profiles to study the profile changes or the transitions, respectively. In particular, experimental designs could be used to disentangle the effects stemming from interventions and naturally occurring development process in explanation skills. However, such a procedure would require a large sample size, particularly in the various groups, to analyze potentially different epistemological belief profiles.

Another potentially important aspect for further research is the intervention type, in particular, the content and the type of the intervention. Studies that examine epistemological belief change (e.g., Rosman et al., 2016; Klopp and Stark, 2022a,b) usually draw on the presentation of divergent information, e.g., in the form of scientific controversies. The existence of such controversies is incompatible with absolutist and multiplicist epistemological beliefs and should induce epistemological doubt that yields a change towards evaluativist beliefs. The results from this study also indicate that the confrontation with scientific methods like explanations may alter epistemological beliefs, too, and may potentially enrich the set of methods used in epistemological belief change research. However, in contrast to presenting controversies, the mechanics of epistemological change are different. In the case of this study, epistemological doubt should arise because some occurrences of epistemological beliefs are incompatible with explanations norms, e.g., the norm to use scientific theories in explanations may be incompatible with a low belief in the justification of theories by the scientific community. Consequently, the presentation of this norm may lead to epistemological doubt. Additionally, learning from advocatory errors represents a particular presentation of the resolution strategies. As learning from errors contains the error and the solution and consists in the reflection of the contrast between the error and the solution (Wagner et al., 2014a,b), it represents a straightforward presentation of the resolution strategies. This way, past experiences with a naïve scientific concept can be analyzed and compared to the new scientific concept resulting in the acquisition of the new concept. At the same time, the old and inadequate epistemological beliefs can be adapted to the new concept, thereby reducing epistemological doubt and finally bringing the epistemological belief change. Thus, working with scientific concepts like explanations and learning from advocatory errors may be a new paradigm to induce and study epistemological change processes.

## Data availability statement

The raw data supporting the conclusions of this article will be made available by the authors on request, without undue reservation.

## Ethics statement

Ethical approval was not required by law for the studies involving humans. The studies were conducted in accordance with the local legislation and institutional requirements. The participants provided their written informed consent to participate in this study.

## Author contributions

EK conceptualized the study, developed the intervention, did the statistical analysis, and wrote, together with TK-W, the manuscript. RS assisted in the development of the materials and supervised the conceptualization, read and commented on the draft of the manuscript, and reviewed the analysis results. All authors contributed to the article and approved the submitted version.

## Footnotes

^1^In this paper, we only deal with the DN model and leave other models of scientific explanation (cf., Woodward, 2014) aside.

^2^The term adequate is related to the notion of productive epistemological beliefs that was introduced by Elby and Hammer (2001), but differs in some ways from the concept of an adequate belief.

^3^Note that we use the terms classes and profiles interchangeably.
