# Supplementary material for: Profiles of epistemological beliefs, knowledge about explanation norms, and explanation skills: changes after an intervention
Source: Front Psychol. 2023 Oct 20;14:1178129. doi: 10.3389/fpsyg.2023.1178129 (PMC10622974; doi:10.3389/fpsyg.2023.1178129)
Supplement: Supplementary file 1 [file Data_Sheet_1.PDF]

Supplementary Material

Profiles of epistemological beliefs, knowledge about explanation norms, and explanation skills: Changes after an intervention

Eric Klopp[*], Theresa Krause-Wichmann, Robin Stark

*** Correspondence:** Eric Klopp, e.klopp@mx.uni-saarland.de

1 Pre- and Posttest scenarios

In the pretest, the scenario featured the case of a student showing bad grades in mathematics and symptoms of performance anxiety. The case also featured details of the family background: The student has an older brother with good mathematics grades and a father with high expectations due to the older brother's good mathematics performance. However, the student could not achieve his brother's grades and thus began to get sick before exams. The collection of theories contained the theory of performance anxiety, the transactional model, an attributional theoretical account of test anxiety, and a learning-theoretical account of performance anxiety. The participants had to recognize that the theory of performance anxiety best fits the situation and mention that they will use this theory for their explanation (Theory mentioned). They then had to link the description of the family background, the described symptoms, and the bad grades to the pertinent elements of the theory of performance anxiety (Theory-evidence-coordination for the explanans and explanandum). The explanation also needed to show the bad grades and symptoms resulting from the family background (Logical correctness). The participants had to note that the transactional model only fit the situation partly and that attributional and learning theoretical accounts of performance anxiety did not fit the situation (Multiperspectivity). Table S1 provides an overview of the rating criteria for the pretest scenario.

In the posttest, the scenario featured a case of a young mathematics teacher who is new to a class and prefers cooperative learning methods. He introduces learning in groups in his class, which, in general, works well. But there is one problematic student who shows disturbing behavior, particularly during group work, and also receives bad grades in mathematics. However, the student only shows this behavior in the young teacher's mathematics class. The teacher seeks advice from the former mathematics teachers in the class. He learns that the problem student did not show disturbing behavior earlier and (also) received good grades. Also, the former teacher applied teacher-centered teaching methods, and the students had to work independently. The collection of theories that the participants received to explain the situation contained the theory of uncertainty and certainty-oriented learners, the theory of instrumental aggression, the frustration-aggression-hypothesis, and the need for achievement theory. The participants had to recognize that the theory of uncertainty and certainty-oriented learners fits best to the situation and had to mention the theory (Theory mentioned). They had to relate the disturbing behavior to the uncertainty-avoidance of the students and the resulting bad grades to the misfit between the student's learning preferences and the young teacher's teaching methods (Theory-evidence-coordination). The explanation had to showcase the disturbing behavior and the bad grades as a result of this misfit (Logical correctness). The participants also had to recognize that the frustration-

aggression-hypotheses partially fit the situation (Multiperspectivity). They had to interpret the misfit between the learner's preferences and the teacher's teaching method as anger-inducing. They had to relate the disturbing behavior to aggression resulting from the frustration (Theory-evidence-coordination). The explanation also had to showcase the disturbing behavior as a result of the frustration (Logical correctness). Furthermore, they had to rule out the theory of instrumental aggression and the need for achievement theory as these did not fit the situation (Multiperspectivity). Table S2 provides an overview of the rating criteria for the pretest scenario.Table S1. Coding criteria and points for EC score in the pretest.

Explanation norm	Rating [points]	Max. points
Theory mentioned: Performance anxiety	0 = Theory is not mentioned<br>1 = Theory is mentioned<br>2 = Theory is explained in detail	2
Theory-evidence-coordination: Explanans performance anxiety	0 = Situation is not mentioned<br>1 = no theory-evidence-coordination<br>2 = basal theory-evidence-coordination<br>3 = detailed theory-evidence-coordination	3
Theory-evidence-coordination: Explanandum, symptoms of performance anxiety	0 = Situation is not mentioned<br>1 = no theory-evidence-coordination<br>2 = basal theory-evidence-coordination<br>3 = detailed theory-evidence-coordination	3
Theory-evidence-coordination: Explanandum, test performance	0 = Situation is not mentioned<br>1 = no theory-evidence-coordination<br>2 = basal theory-evidence-coordination<br>3 = detailed theory-evidence-coordination	3
Logically correct deduction for performance anxiety	0 = incorrect<br>1 = correct	1
Theory mentioned: Transactional model	0 = Theory is not mentioned<br>1 = Theory is mentioned<br>2 = Theory is explained in detail	2
Theory-evidence-coordination: Explanans parental behavior	0 = Situation is not mentioned<br>1 = no theory-evidence-coordination<br>2 = basal theory-evidence-coordination<br>3 = detailed theory-evidence-coordination	3
Theory-evidence-coordination: Explanandum performance anxiety	0 = mentioned<br>1 = not mentioned	1
Logically correct deduction for transactional model	0 = incorrect<br>1 = correct	1

Explanation norm	Rating [points]	Max. points
Multiperspectivity: Rule out attributional and learning theoretical accounts	0 = not mentioned<br>1 = wrongly excluded<br>2 = only mentioned<br>3 = superficially justified exclusion<br>4 = justified exclusion	4

Table S2. Coding criteria and points for EC score in the posttest.

Explanation norm	Rating [points]	Max. points
Theory mentioned: Learner types	0 = Theory is not mentioned<br>1 = Theory is mentioned<br>2 = Theory is explained in detail	2
Theory-evidence-coordination: Explanans learner types	0 = Situation is not mentioned<br>1 = no theory-evidence-coordination<br>2 = basal theory-evidence-coordination<br>3 = detailed theory-evidence-coordination	3
Theory-evidence-coordination: Explanandum, aggression	0 = Situation is not mentioned<br>1 = no theory-evidence-coordination<br>2 = basal theory-evidence-coordination<br>3 = detailed theory-evidence-coordination	3
Theory-evidence-coordination: Explanandum, performance	0 = Situation is not mentioned<br>1 = no theory-evidence-coordination<br>2 = basal theory-evidence-coordination<br>3 = detailed theory-evidence-coordination	3
Logically correct deduction for aggression and performance	0 = incorrect<br>1 = correct	1
Theory mentioned: Frustration-aggression-hypothesis	0 = Theory is not mentioned<br>1 = Theory is mentioned<br>2 = Theory is explained in detail	2
Theory-evidence-coordination: Explanans, frustration	0 = Situation is not mentioned<br>1 = no theory-evidence-coordination<br>2 = basal theory-evidence-coordination<br>3 = detailed theory-evidence-coordination	3
Theory-evidence-coordination: Explanans, school context	0 = Situation is not mentioned<br>1 = no theory-evidence-coordination<br>2 = basal theory-evidence-coordination<br>3 = detailed theory-evidence-coordination	3

Explanation norm	Rating [points]	Max. points
Theory-evidence-coordination: Explanandum, aggression	0 = Aggression not mentioned<br>1 = Aggression mentioned	1
Theory-evidence-coordination: Explanandum, aggression, shifting	0 = Situation is not mentioned<br>1 = no theory-evidence-coordination<br>2 = basal theory-evidence-coordination<br>3 = detailed theory-evidence-coordination	3
Logically correct deduction for aggression and shifting	0 = incorrect<br>1 = correct	1
Multiperspectivity: Rule out instrumental aggression and need for achievement	0 = not mentioned<br>1 = wrongly excluded<br>2 = only mentioned<br>3 = superficially justified exclusion<br>4 = justified exclusion	4

2 Epistemological belief scale items

Table S3

No.	Item
Personal justification	
PJ1	What can be classified as a scientific fact depends on one's personal opinions.
PJ2	One can have different opinions concerning scientific contents because completely correct answers don't exist.
PJ3	Scientific knowledge is only the scientist's personal opinion because there are no facts.
PJ4	Scientists only sell their own views as scientific findings.
PJ5	Scientific findings originate mainly from the respective scientist's opinions.
Justification by authority	
JA1	If a scientist considers something to be correct, I believe it.
JA2	What you can find in scientific texts and books is correct.
JA3	If a scientist tells me something, I believe it.
JA4	If I read something that is based on scientific research, I know that it is true.
JA5	I believe statements which are based on scientific research to be correct.
Justification by multiple sources	
JS1	In order to trust scientific statements, different sources have to be verified.
JS2	In order to detect incorrect statements in a scientific text, it is important to use several sources.
JS3	I cannot be sure about a scientific statement as long as I haven't checked at least one other source.
JS4	One source alone is never enough to decide if something is scientifically correct.
JS5	In order to decide whether a scientific statement is correct, I have to check if it is in agreement with other information.

No.	Item
Justification by the scientific community	
JC1	Only published findings can be classified as knowledge.
JC2	A statement/assertion has to be verified by other scientists.
JC3	New findings have to be communicated to others in order to become valid.
JC4	Findings can only be considered as knowledge if others validate them.
JC5	Knowledge is generated through discourse in the scientific community.
Certainty of knowledge	
CK1	There are scientific findings which will always be valid.
CK2	In the end scientists can get to the truth.
CK3	There are irrevocable truths.
CK4	Most of our knowledge won't become obsolete in the future.
CK5	In science, there is a firm core of knowledge.
Reflective nature of knowledge	
RN1	Knowledge will develop if we deal with it in a critical way.
RN2	The previous knowledge is often questioned due to new findings.
RN3	The work with new contents reveals already known facts in a different light.
RN4	One can see problems from a new perspective after intense reflections.
RN5	The evaluation/assessment of knowledge changes once there are new findings.

3 Detailed description of the training intervention

The training intervention consisted of an information sheet and four paper-and-pencil learning units. Figure 2 in the main text provides an overview of the structure of the training intervention. Before the first learning unit, the participants received a worksheet that introduced them to the concept of scientific explanation and the DN model. The worksheet contained several worked examples (cf., Klopp & Stark, 2018). This worksheet aimed to introduce the participants to the concept of scientific explanation. Additionally, the most important information norms were introduced. These norms were: using current scientific theories and empirical results (in contrast to everyday psychological or subjective theories) and multiperspectivity. This was followed by a simple and well-structured example for a scientific explanation. The example featured the case of a student having parents with an authoritative parenting style and being a bullying victim. The scientific law consisted in the empirical results that children with authoritative parents will likely be bullying victims later on (Lereya, Samara, &Wolke, 2013). Afterward, the participants were asked to work out which features of explanations are included in the example. The participants received a sample solution. A second, more complex example using Atkinson's (1957) risk choice model followed. In contrast to the first example, this one featured multiperspectivity and discussed possible alternative explanations and theories that did not fit the situation. After this example, the participants were asked again to work out which features of explanations were included in the example and then received a sample solution. Finally, the participants were presented with a case of cognitive dissonance after a forced choice. The students were asked to work out an explanation using Festinger's (2012) cognitive dissonance theory. The example was taken from Klopp and Stark (2018), and the participants received a sample explanation.

After the worksheet, the learning units followed. These learning units draw on the concept of learning from advocatory errors. To ensure the relevance of the content for the learners and to foster the identification of the learners with the featured character that commits an error, each learning unit is embedded in a story featuring a young teacher who has observed an authentic school situation and now

wants to provide a scientific explanation. The story is framed as follows: The young teacher provides his explanation to an experienced school psychologist and a board of colleagues in a meeting. A learning unit consists of four parts. In the first part, the young teacher's erroneous explanation is presented to the participants. In the frame of the story, the young teacher presents his explanation. Then the participants were instructed to work out how the young teacher justified his explanation, i.e., they should elaborate on which theory the young teacher used for his explanation. After that, the participants were instructed to work out how the young teacher underpinned his explanation, i.e., they should elaborate on his choice of theories and the theory-evidence-coordination. Finally, the participants had to elaborate on how they had explained the situation.

In the second part, the school psychologist analyzes the young teacher's explanation. The explanation contained at least two errors, i.e., violations of explanation norms that the school psychologist scrutinized. The school psychologist explained the norm violations. These violations of explanation norms are summarized in Table S4. In the third part, the participants received a multiple-choice repetition test about the presented norm violations. Each multiple-choice question had three answer options. After the participants completed the test, the correct answer was presented with the instruction to compare their solution with the sample solution. In the story, the test is framed by the question of a colleague who asked the young teacher for the definitions of the norm violations.

In the third part, the school psychologist presented his explanation of the situation. This started with a collection of possible theories. Then the school psychologist outlined the actual explanation, which also entailed modeling the theory-evidence-coordination. This part served as the contrast between the earlier presented erroneous explanation and the correct explanation. Afterward, the school psychologist presented strategies for avoiding the presented violations of explanation norms.

In the fourth and last part, the participants were instructed to reflect on how they had avoided the violations of explanation norms featured in the young teacher's explanation. This was an open-ended question, and no solution was provided. The contents of the learning units, i.e., the topics in which they were framed, are summarized in Table S4.

Table S4. Contents of the training intervention.

Learning unit	Violated explanation norm	Framing
1	Circular argument<br>Combination of circular argument and everyday theory (fundamental attribution error)<br>Use of an inappropriate theory	Bullying
2	Premature closure<br>Lack of multiperspectivity<br>Use of an outdated theory	Group processes
3	Everyday theory (fundamental attribution error<br>Use of a subjective theory<br>Combination of inappropriate and subjective theory	Learning motivation
4	Superficial interpretation of empirical results<br>Superficial interpretation of outdated results<br>Combination of premature closure and lack of multiperspectivity	Disciplinary problems
